# Supplementary material for: Optimizing next-generation RSV prevention in Mali: A cost-effectiveness analysis of pediatric vaccination, maternal vaccination, and extended half-life monoclonal antibody immunoprophylaxis
Source: PLOS Glob Public Health. 2023 May 5;3(5):e0001432. doi: 10.1371/journal.pgph.0001432 (PMC10162555; doi:10.1371/journal.pgph.0001432)
Supplement: S1 Text — Methods: Supplement Table A: Summary of input parameters used for the base case analysis; Supplement Figure A: Health outcome tree for monthly birth cohorts followed until three years; Supplement Figure B: RSV attack rate probability sampling distributions by age in months; Supplement Figure C: RSV prevention product administration and protection schedules; Supplement Figure D: Hospitalization rate given RSV-LRTI probability sampling distributions by age in months; Supplement Figure E: Attributable fatality rate probability sampling distributions by age in months; Supplement Figure F: Comparison of case-fatality rates among hospitalized infants with RSV-LRTI in LMICs and Mali; Supplement Figure G: Other input parameter probability sampling distributions. Results: Supplement Table B: Budget impact and cost-effectiveness analysis by payer perspective; Supplement Figure H: Optimal strategy across willingness to pay from both the government and donor perspective; Supplement Figure I: Sensitivity analysis on optimal strategy across changing efficacy of the pediatric vaccine at 10/14 weeks; Supplement Figure J: Alternate scenario analysis where the maternal vaccine has higher coverage and is delivered seasonally as opposed to year-round. (DOCX) [file pgph.0001432.s001.docx]

**Online Supplement**

Optimizing next-generation RSV prevention in Mali: a cost-effectiveness analysis of pediatric vaccination, maternal vaccination, and extended half-life monoclonal antibody immunoprophylaxis

Contents:

Methods

1. Supplement Table A: Summary of input parameters used for the base case analysis
2. Supplement Fig A: Health outcome tree for monthly birth cohorts followed until three years
3. Supplement Fig B: RSV attack rate probability sampling distributions by age in months
4. Supplement Fig C: RSV prevention product administration and protection schedules
5. Supplement Fig D: Hospitalization rate given RSV-LRTI probability sampling distributions by age in months
6. Supplement Fig E: Attributable fatality rate probability sampling distributions by age in months
7. Supplement Fig F: Comparison of case-fatality rates among hospitalized infants with RSV-LRTI in LMICs and Mali
8. Supplement Fig G: Other input parameter probability sampling distributions

Results

1. Supplement Table B: Budget impact and cost-effectiveness analysis by payer perspective
2. Supplement Fig H: Optimal strategy across willingness to pay from both the government and donor perspective
3. Supplement Fig I: Sensitivity analysis on optimal strategy across changing efficacy of the pediatric vaccine at 10/14 weeks
4. Supplement Fig J: Alternate scenario analysis where the maternal vaccine has higher coverage and is delivered seasonally as opposed to year-round.

**Supplement Table A:** Summary of input parameters used for the base case analysis

|  | **Value (95% Confidence Interval, when used)** | **Rationale** |
| --- | --- | --- |
| **Epidemiologic parameters** |  |  |
| RSV attack rate | Age-specific, see Supplement Figure 4. | We started with community-based RSV incidence rates per 1000 person-years in Mali for children in the first six months of life [3]. Incidence varies by infant age as well as calendar month. Incidence rates for Mali were extrapolated out to 36 months based on the linear decline in the RSV incidence rates described for low-income countries between 6 months and 24 months, and between 24 and 36 months [5]. |
| Probability of LRTI given RSV | 0.13 (0.11, 0.17) | All infants with pneumonia episodes occurring between October 2012 and May 2013 were selected to be tested for RSV in the Mali incidence study, whereas only 48.9% of the infants with influenza-like-illness without pneumonia were tested [3]. To account for the oversampling among infants with pneumonia, we calculate the probability of LRTI given RSV as the proportion of RSV cases with pneumonia reported in the trial adjusted to match the proportion of infants with influenza-like-illness but without pneumonia who were tested for RSV. |
| Probability of inpatient care given RSV-LRTI by age | Age-specific, see Supplement Figure 5. | We applied an age-specific gradient for hospitalization rates from the Gambia [6] to the Mali-specific rate of hospitalization given RSV LRTI [3]. Starting at 0·30 in the first month of life, these rates decline to 0·12 by the 36^th^ month of life. All infants with pneumonia who did not receive inpatient care received outpatient care in the Mali community incidence study [3]. The probability of outpatient care given RSV-LRTI is therefore calculated as 1- probability of inpatient care given RSV-LRTI. |
| Case fatality rate among those who received inpatient care given RSV-LRTI by age | Age-specific, see Supplement Figure 6. | We used age-specific hospital case fatality rates for RSV LRTI in LMICs [7]. The probability of death given inpatient care is 0·27 in the first month of life and declines to 0·008 by the 36^th^ month of life. We assume 49% of RSV-LRTI deaths occur outside the hospital [6]. |
| Disability weight inpatient RSV-LRTI | 0.13 (0.10, 0.17) | The 2017 Institute for Health Metrics and Evaluation Global Burden of Disease disability weight for an acute episode of a severe LRTI, used to calculate DALYs [8]. |
| Disability weight outpatient RSV-LRTI | 0.05 (0.04, 0.07) | The 2017 Institute for Health Metrics and Evaluation Global Burden of Disease disability weight for an acute episode of a moderate LRTI, used to calculate DALYs [8] |
| Duration of RSV illness (days) | 8.5 (7, 10) | The average duration of illness for episodes of lower-respiratory tract infections based on health facility data [9]. |
| **Demographic parameters** |  |  |
| Crude birth rate (per 1,000) | 42 | The World Bank crude birth rate per 1,000 total population for Mali in 2017 [1]. |
| Total country population | 18,540,000 | The World Bank total population estimate for Mali in 2017 [1]. |
| Number of infants in each birth cohort | 59,734 – 66,134 | The number of infants in each monthly birth cohort was calculated by first multiplying the crude birth rate by the total country population for Mali to estimate the number of infants born in one year. The total number of births for each month was assigned based on the number of days in each month. |
| Life expectancy at birth | 58 | Average 2017 life expectancy at birth for Mali, used to calculate DALYs [1]. |
| **Economic parameters** |  |  |
| Inpatient care costs (USD) | 118.57 (92.20, 144.68) | Average cost of medical care for infants less than six months with confirmed RSV illness who received inpatient care. Costs are inclusive of outpatient services also acquired by this group [10]. We assume 53% of severe RSV-LRTI episodes do not receive inpatient care [6]. |
| Outpatient care costs (USD) | 6.56 (5.44, 7.66) | Average cost of medical care for infants less than six months with confirmed RSV illness who received outpatient care services only [10]. |
| Administration cost of adding a new product to a childhood immunization visit (USD) | 0.67 | The mean incremental cost of adding one product to the established immunization schedule in low-income countries, equal to $0.67 [11]. |

**Supplement Fig A:** Health outcome tree for monthly birth cohorts followed until three years. Ellipses indicate symmetrical sub-trees in the intervention arms, with probability of RSV LRTI conditional on the intervention and all other features identical.

**Supplement Fig B:** RSV attack rate probability sampling distributions by month of age. The red line represents the point estimate**.**

**Supplement Fig C:** RSV prevention product administration and protection schedules.

**Supplement Fig D:** Hospitalization rate given RSV-LRTI probability sampling distributions by age. The red line represents the point estimate.

**Supplement Fig E:** Attributable fatality rate given RSV-LRTI hospitalization probability sampling distributions by month of age. The red line represents the point estimate.

**Supplement Fig F:** Comparison of case-fatality rates (CFR) in the first six months of life among hospitalized infants with RSV-LRTI in LMICs [6], and Mali [12]. T-bars indicate the 95% confidence intervals.

**Supplement Fig G:** Other input parameter probability sampling distributions. The red line represents the point estimate.

**Supplement Table B:** Budget impact and cost-effectiveness analysis by payer perspective

|  | DALYs averted compared to status quo | Medical costs | Donor costs | Incremental cost-effectiveness ratio, donor perspective | Government costs | Incremental cost-effectiveness ratio, government perspective | Societal costs | Incremental cost-effectiveness ratio, societal perspective |
| --- | --- | --- | --- | --- | --- | --- | --- | --- |
| Status quo | --- | $1249382 (742201, 1546908) | --- | --- | $1249382 (742201, 1546908) | --- | $1249382 (742201, 1546908) | --- |
| Maternal vaccine | 347 (140, 569) | $1176679 (699904, 1455074) | $269954 | *Weakly dominated* | $1470194 (977962, 1719329) | *Weakly dominated* | $1740148 (1263373, 2018543) | *Weakly dominated* |
| Long-acting monoclonal antibody | 878 (369, 1393) | $1048241 (624702, 1288465) | $347339 | $396 (50, 928) | $1425895 (989196, 1652832) | $201 (94, 664) | $1773233 (1349695, 2013457) | $597 (354, 1621) |
| Pediatric vaccine at 10 & 14 weeks | 1247 (548, 1848) | $817669 (486934, 1018490) | $960114 | *Weakly dominated* | $1861579 (1523004, 2045810) | *Weakly dominated* | $2821693 (2490958, 3022514) | *Weakly dominated* |
| Pediatric vaccine at 6 & 7 months | 558 (245, 805) | $1006701 (596633, 1261419) | $872831 | *Strongly dominated* | $1955710 (1532775, 2198145) | *Strongly dominated* | $2828541 (2418472, 3083259) | *Strongly dominated* |
| Maternal vaccine plus sequential pediatric vaccine at 10 & 14 weeks | 1594 (705, 2387) | $744966 (445934, 924936) | $1230068 | *Weakly dominated* | $2082391 (1774726, 2245343) | *Weakly dominated* | $3312459 (3013427, 3492429) | *Weakly dominated* |
| Maternal vaccine plus sequential pediatric vaccine at 6 & 7 months | 906 (400, 1343) | $933998 (554562, 1167781) | $1142785 | *Strongly dominated* | $2176522 (1784383, 2397700) | *Strongly dominated* | $3319307 (2939871, 3553090) | *Strongly dominated* |
| Long-acting monoclonal antibody plus sequential pediatric vaccine at 6 & 7 months | 1436 (630, 2155) | $805560 (480774, 1002645) | $1220169 | *Strongly dominated* | $2132223 (1795509, 2319766) | *Strongly dominated* | $3352392 (3027606, 3549477) | *Strongly dominated* |
| Long-acting monoclonal antibody plus sequential pediatric vaccine at 10 & 14 weeks | 1947 (855, 2936) | $662438 (398664, 819499) | $1307452 | $898 (605, 2034) | $2084002 (1812017, 2225676) | $615 (380, 1660) | $3391454 (3127680, 3548516) | $1514 (999, 3705) |

**Supplement Fig H:** **A)** Optimal strategy across willingness to pay from the government perspective. **B)** Optimal strategy across willingness to pay from the donor perspective.

**Supplement Fig I: A)** Sensitivity analysis on optimal strategy across changing efficacy of the pediatric vaccine when administered at 10/14 weeks. **B)** Sensitivity analysis on optimal strategy across changing efficacy of the pediatric vaccine when administered at 10/14 weeks as part of a combination strategy in which either maternal vaccine or monoclonal antibodies were administered prior to the pediatric vaccine.

**Supplement Fig J:** Alternate scenario analysis for the optimal strategy where the maternal vaccine has higher coverage (89.1%) and is delivered seasonally as opposed to year-round. In this scenario, only our assumptions for maternal vaccine were changed, all other strategies remained the same as in the base case. **A)** Probability each RSV LRTI prevention strategy is optimal given a product price per dose and willingness-to-pay of $891, equal to the per capita GDP of Mali. For comparison, prices for Measles-Rubella (MR), DPT Hep B Hib (Penta), and Typhoid (TCV) vaccine are indicated by the dashed lines. **B)** Probability each RSV LRTI prevention strategy is optimal given a societal WTP to avert disability-adjusted life-years and an intervention product price of $1.00. Although they are no longer considered standard for WTP values, benchmarks of 1 X GDP and 3 X GDP per capita are drawn here for comparison. The competing scenarios are status quo, extended half-life monoclonal antibody (mAb), maternal vaccine (mVax), pediatric vaccine (pVax) administered at 10 and 14 weeks, pVax administered at 6 & 7 months, as well as combinations mAb + pVax and mVax + pVax. Strategies not visible on the plot are never the optimal choice and therefore remain at y = 0.

**References:**

1. Mali Data. 2020 [cited 23 Apr 2020]. Available: https://data.worldbank.org/country/mali

2. Mali - Climatology | Climate Change Knowledge Portal. [cited 30 Nov 2022]. Available: https://climateknowledgeportal.worldbank.org/country/mali/climate-data-historical

3. Buchwald AG, Tamboura B, Tennant SM, Haidara FC, Coulibaly F, Doumbia M, et al. Epidemiology, Risk Factors, and Outcomes of Respiratory Syncytial Virus Infections in Newborns in Bamako, Mali. Clin Infect Dis. 2020;70: 59–66. doi:10.1093/cid/ciz157 10.1093/cid/ciz157.

4. WorldClim. [cited 30 Nov 2022]. Available: https://worldclim.org/

5. Li X, Willem L, Antillon M, Bilcke J, Jit M, Beutels P. Health and economic burden of respiratory syncytial virus (RSV) disease and the cost-effectiveness of potential interventions against RSV among children under 5 years in 72 Gavi-eligible countries. BMC Med. 2020;18. doi:10.1186/s12916-020-01537-6

6. Shi T, McAllister DA, O’Brien KL, Simoes EAF, Madhi SA, Gessner BD, et al. Global, regional, and national disease burden estimates of acute lower respiratory infections due to respiratory syncytial virus in young children in 2015: a systematic review and modelling study. The Lancet. 2017;390: 946–958. doi:10.1016/S0140-6736(17)30938-8

7. Li X, Willem L, Antillon M, Bilcke J, Jit M, Beutels P. Health and economic burden of respiratory syncytial virus (RSV) disease and the cost-effectiveness of potential interventions against RSV among children under 5 years in 72 Gavi-eligible countries. BMC Med. 2020/04/07. 2020;18: 82. doi:10.1186/s12916-020-01537-6

8. Global Burden of Disease Study 2017 (GBD 2017) Disability Weights | GHDx. [cited 30 Apr 2020]. Available: http://ghdx.healthdata.org/record/ihme-data/gbd-2017-disability-weights

9. Mathers CD, Vos T, Lopez AD, Salomon J, Ezzati M. National Burden of Disease Studies: A Health, Practical Guide. Edition 2.0. Global Program on Evidence for Health Policy. Geneva: World Health Organization; 2001. Available: https://www.who.int/healthinfo/nationalburdenofdiseasemanual.pdf

10. Orenstein EW, Orenstein LA, Diarra K, Djiteye M, Sidibe D, Haidara FC, et al. Cost-effectiveness of maternal influenza immunization in Bamako, Mali: A decision analysis. PLoS One. 2017;12. doi:10.1371/journal.pone.0171499 10.1371/journal.pone.0171499. eCollection 2017.

11. Baral R, Higgins D, Regan K, Pecenka C. Impact and cost-effectiveness of potential interventions against infant respiratory syncytial virus (RSV) in 131 low-income and middle-income countries using a static cohort model. BMJ Open. 2021;11. doi:10.1136/bmjopen-2020-046563

12. O’Brien KL, Baggett HC, Brooks WA, Feikin DR, Hammitt LL, Higdon MM, et al. Causes of severe pneumonia requiring hospital admission in children without HIV infection from Africa and Asia: the PERCH multi-country case-control study. The Lancet. 2019;394: 757–779. doi:10.1016/S0140-6736(19)30721-4
